# Association between smoking and total energy expenditure in a multi-country study

**DOI:** 10.1186/1743-7075-11-48

**Published:** 2014-10-04

**Authors:** Semira Gonseth, Lara Dugas, Barathi Viswanathan, Terrence Forrester, Vicki Lambert, Jacob Plange-Rhule, Ramon Durazo-Arvizu, Amy Luke, Dale A Schoeller, Pascal Bovet

**Affiliations:** Department of Epidemiology and Biostatistics, University of California, San Francisco, CA 94158 USA; Institute of Social and Preventive Medicine, Lausanne University Hospital, Lausanne, Switzerland; Department of Public Health Sciences, Stritch School of Medicine, Loyola University Chicago, Maywood, IL USA; NCD Section, Ministry of Health, Victoria, Republic of Seychelles; Tropical Medicine Research Institute, University of the West Indies, Kingston, Jamaica; Research Unit for Exercise Science and Sports Medicine, University of Cape Town, Cape Town, South Africa; Kwame Nkrumah University of Science and Technology, Kumasi, Ghana; Department of Nutritional Sciences, University of Wisconsin, Madison, WI USA

**Keywords:** Smoking, Doubly labeled water, Total energy expenditure, Physical activity, Accelerometer, Body mass index, Body weight

## Abstract

**Background:**

The association between smoking and total energy expenditure (TEE) is still controversial. We examined this association in a multi-country study where TEE was measured in a subset of participants by the doubly labeled water (DLW) method, the gold standard for this measurement.

**Methods:**

This study includes 236 participants from five different African origin populations who underwent DLW measurements and had complete data on the main covariates of interest. Self-reported smoking status was categorized as either light (<7 cig/day) or high (≥7 cig/day). Lean body mass was assessed by deuterium dilution and physical activity (PA) by accelerometry.

**Results:**

The prevalence of smoking was 55% in men and 16% in women with a median of 6.5 cigarettes/day. There was a trend toward lower BMI in smokers than non-smokers (not statistically significant). TEE was strongly correlated with fat-free mass (men: 0.70; women: 0.79) and with body weight (0.59 in both sexes). Using linear regression and adjusting for body weight, study site, age, PA, alcohol intake and occupation, TEE was larger in high smokers than in never smokers among men (difference of 298 kcal/day, *p* = 0.045) but not among women (162 kcal/day, *p* = 0.170). The association became slightly weaker in men (254 kcal/day, *p =* 0.058) and disappeared in women (−76 kcal/day, *p =* 0.380) when adjusting for fat-free mass instead of body weight.

**Conclusion:**

There was an association between smoking and TEE among men. However, the lack of an association among women, which may be partly related to the small number of smoking women, also suggests a role of unaccounted confounding factors.

## Introduction

Tobacco use is the leading avoidable cause of death worldwide [1]. The belief that smoking may be a weight control agent constitutes a reason to initiate or to pursue smoking, especially among women and adolescents [2–9]. Nevertheless, findings on the effects of smoking on body weight are inconsistent in the literature. Body weight was shown to be lower in smokers compared to nonsmokers in many population-based studies conducted a few decades ago [10–13], however, several recent cohort studies [14, 15] report no body weight lowering effects. A recent Mendelian randomization analysis suggested that smoking causes lower body mass index (BMI) [16]. It is therefore important to update the current literature investigating the association between smoking and body weight, in particular with regards to claims about the benefits of smoking related to weight control, which may undermine national tobacco control programs.

Two broad mechanisms may underlie an effect of smoking on body weight. First it is reported that smoking increases total energy expenditure (TEE) through increasing the resting metabolic rate (RMR) and/or decreasing energy intake as a result of suppressing appetite [17, 18]. A number of experimental studies showed that a moderate increase in energy expenditure occurred for short periods of time right after cigarette smoking [19–22], although no effect was found in another study [23]. More recently, two cross-sectional studies examined the association between smoking and TEE in smokers vs. non smokers using the doubly labeled water (DLW) technique, the gold standard to assess TEE [24]. Warwick *et al.* found no association [25] but the study relied on a very small sample size (i.e., 21 participants). The second study, by Bradley et al. involved 304 participants and also did not find an association between TEE and smoking but smoking was not categorized by the intensity of the exposure, e.g. as light or heavy smokers [17, 25].

In this paper, we examine the association between smoking, BMI and TEE measured with DLW in a subset of participants of an ongoing study in several populations of African origin (Modeling the Epidemiological Transition Study—METS) [26]. We hypothesized that smokers would have a higher TEE and a lower BMI compared to never smokers.

## Methods

METS is an ongoing prospective cohort study investigating the contribution of physical activity (PA) to obesity, diabetes and hypertension [26]. Briefly, METS is being conducted in urban South Africa (Khayelitsha, a large township adjacent to the city of Cape Town), the Seychelles (the main island of Mahé), urban Jamaica (city of Kingston), rural Ghana (villages near Kumasi) and metropolitan Chicago (suburban community of Maywood).

At each site, 500 adults aged 25–45 years with similar proportions of males and females were recruited and baseline data collected during 2010–2011, of which ca. 75 participated in the DLW measurement of TEE. A description of the study protocol has been published elsewhere [26]. The vast majority of the population in each study site was of predominantly African descent and persons from non African descent were not selected in Seychelles where around 20% of the total population is of Caucasian, Indian or Chinese descent. We selected the study sites to represent a broad range of social and economic development according to the United Nations Human Development Index (HDI) 2010 definition: Ghana is a low HDI country, South Africa is a middle HDI country, Jamaica and the Seychelles are high HDI countries, and the US is a very high HDI country [27].

We excluded individuals with an active infectious disease (including HIV-positive individuals), pregnant or lactating women, as well as persons with conditions preventing normal physical activities, e.g. lower extremity disability. In Ghana, we generated a simple random sample for the age-range of the study based on the population census for the rural town of Nkwantakese. In both Seychelles and South Africa, we generated sex- and age-stratified random samples based on their respective national censuses. In Kingston, Jamaica, we sampled districts at random; we fixed a point in each district (e.g., the north-west corner) from which we carried out door-to-door recruitment. Similarly, in Maywood, IL, USA, we randomized all city blocks in the community and we conducted door-to-door recruitment. Thus the cohorts enrolled were representative of their respective communities and cannot be considered as nationally representative samples.

The Institutional Review Board of each participating institution approved METS (25). A written and informed consent was obtained from each participant. Participants from Ghana were not included in the present analysis because there were no current daily smokers. Similarly, occasional smokers and ex smokers were also excluded from the analysis. The final sample included 236 never or current smokers with complete data on energy expenditure and PA at the baseline visit in 2010–2011.

### Body composition

We made all measurements at outpatient clinics located in the communities. Weight and height were measured using the same model calibrated instruments at all sites. In all study sites, weight was measured with precision electronic scales (Seca 770, Seca, Hamburg, Germany) to the nearest 0.1 kg and height was measured using a fixed stadiometer to the nearest 0.1 cm, with the participants wearing light clothing and no shoes. BMI was calculated as weight divided by height squared (kg/m^2^). Total body water (TBW) was obtained from the dilution of deuterium and ^18^O. Fat-free mass (FFM) was derived as FFM = TBW/0.732 [28].

### TEE

TEE was measured using the DLW method using a standard procedure [24]. In brief, participants were given an oral dose of DLW adjusted to participants’ body weight in order to contain approximately 1.8 g of 10% H_2_^18^O and 0.12 g 99.9% ^2^H_2_O per kg body water. Spot urine samples were collected prior to dosing and 1, 3 and 4 hours follow isotope administration and a final urine sample was collected 8 days later. Isotope ratio mass spectrometric analyses of the DLW urine samples were carried out at the Stable Isotope Core Laboratory at the University of Wisconsin, Madison, USA. The CO_2_ production was calculated using equation 6.6 of the International Atomic Energy Agency technical guidelines [29] and energy expenditure was calculated using the modified Weir equation using the site specific average respiratory exchange ratio equivalent estimated from the diet [30].

### Physical activity

PA was measured using Actical accelerometers (Phillips Respironics, Bend, OR, USA). A combination of amplitude and frequency of motions produce electrical signals that are correlated to the intensity of a participant’s PA. Participants wore the accelerometer for 8 days. Records were considered for all days with 10 or more hours of wear time during day time [31]. PA in this study corresponds to total activity counts divided by total wear time as an overall measure of average PA intensity (mean counts per minute). This technique has good reliability [26].

### Smoking status

Smoking was assessed by an administered structured questionnaire. Current smokers refer to participants smoking at least one cigarette per day and never smokers refer to participants who reported to have never smoked. We dichotomized smoking as “high” for smoking ≥7 cigarettes per day—which is the median of cigarettes smoked per day among all smokers—and “light” for smoking 1–6 cigarettes per day. Questions on participants’ occupation were based on the Core Welfare Indicators Questionnaire from the World Bank. Alcohol consumption refers to the reported number of standard alcohol drinks consumed in a typical weekend.

### Statistical analyses

Differences in continuous variables were tested with the Student t test for men and women separately. The association between smoking and TEE was assessed with crude correlation analysis (coefficient of correlation: r) and with multivariate linear regression (regression coefficient: β). In linear regression analysis, we examined the contributions of different covariates, including: BMI, FFM, age, PA (all as continuous variables); alcohol (as a discrete variable); sex, manual occupation and study sites (as categorical variables). Because of their skewed distribution, alcohol intake and PA were log transformed. All analyses were done on the subset of 236 participants who had complete data on all variables of interest. Statistical significance was set at 0.05. Analyses were done using Stata 13 and R 3.0.2.

## Results

The main characteristics of the 236 participants (58% women, mean age 33.8 years) are reported in Table 1. Current tobacco use was found in 55% of men (mean of 6 cigarettes per day) and in 16% of women (mean of 10 cigarettes per day). The numbers of smokers were highest in the USA and in South Africa and substantially lower in Jamaica and in the Seychelles. BMI tended to be lower in current smokers than in never smokers in men (24.6 and 25.4 [kg/m2] and in women (28.8 and 30.9 [kg/m2]), but differences did not reach statistical significance: p >0.05). Total energy expenditure measured using the DLW method was slightly higher in current smokers than in never smokers in both sexes when analysis was not adjusted for potential confounders (not statistically significant difference of 183 and 95 kcal/day in men and women, respectively). PA tended to be higher in current smokers than in never smokers both among men and women, but difference did not reach statistical significance (230 and 123 [counts/min]) and women (218 and 127 [counts/min]); p > 0.05. This finding was consistent with a higher proportion of manual workers among smokers than never smokers.

**Table 1:** **Participants’ characteristics according to smoking status and sex**

	Men (n = 104)		Women (n = 132)	
	Smoker	Never smoker	Smoker	Never smoker
Participants (n,%)	57 (55%)	47 (45%)	21 (16%)	111 (85%)
Age (years)	33.9 (5.7)	33.0 (5.8)	33.6 (6.2)	33.9 (5.7)
Weight (kg)	75.6 (24.7)	77.3(16.8)	77.8 (22.6)	81.9 (21.4)
Height (cm)	174.6 (7.0)	174.2 (7.0)	164.8 (8.3)	162.7 (5.5)
Fat-free mass (kg)	41.1 (8.3)	41.1 (6.5)	34.9 (4.4)	33.2 (5.7)
Body mass index (kg/m^2^)	24.6 (7.4)	25.4 (4.7)	28.8 (8.6)	30.9 (7.8)
Total energy expenditure (kcal/d)	2830 (710)	2647 (474)	2335 (245)	2240 (420)
Cigarettes per day (n)	6.2 (3.7)		9.8 (9.7)	
Physical activity (mean counts/min)	230 (270)	123 (128)	218 (225)	127 (225)
Manual occupation (%)	81%	66%	57%	50%
Alcohol intake (drinks per week end)	4.5 (3.7)	2.4 (2.9)	2.9 (3.3)	0.9 (1.8)
USA (n,%)	23 (70%)	10 (30%)	12 (40%)	18 (60%)
South Africa (n,%)	18 (67%)	9 (33%)	6 (14%)	37 (86%)
Jamaica (n,%)	8 (36%)	14 (64%)	2 (8%)	24 92%)
Seychelles (n,%)	8 (36%)	14 (64%)	1 (3%)	32 (97%)

Differences in body mass index and physical activity between in smokers vs. non smokers are not statistically significant.

Unless specified otherwise, values are means and standard deviations.

TEE and FFM were highly correlated (correlation coefficient [r] = 0.70 in men and 0.79 in women, Table 2). TEE was weakly correlated with the number of smoked cigarettes per day (r = 0.14 in men and 0.12 in women). TEE was also weakly correlated with total PA measured by accelerometry (r = 0.09 in men and 0.21 in women) and with a manual vs. non-manual occupation (r = 0.17 in men and 0.20 in women). As expected, high correlation coefficients were observed between weight and FFM (r = 0.88 in men and 0.73 in women); weight and BMI (r = 0.96 in men and 0.97 in women); and FFM and BMI (r = 0.80 in men and 0.63 in women).

**Table 2:** **Pairwise correlation coefficients between total energy expenditure (TEE) and selected variables**

		TEE	Fat-free mass	Weight	Height	BMI	Cig/day	PA	Alcohol
Fat-free mass	M	0.70***							
	F	0.79***							
Weight	M	0.59***	0.88***						
	F	0.59***	0.73***						
Height	M	0.31**	0.55***	0.44***					
	F	0.19*	0.37***	0.15					
BMI	M	0.55***	0.80***	0.96***	0.18				
	F	0.54***	0.63***	0.97***	−0.10				
Cig/day	M	0.14	0.02	0.01	0.07	−0.02			
	F	0.12	0.19*	−0.09	0.06	−0.11			
PA	M	0.09	0.02	0.01	0.05	−0.01	0.26		
	F	0.21*	0.16	0.08	0.21*	0.04	0.21		
Manual	M	0.17*	0.01	0.01	−0.08	0.03	0.13	0.11	
	F	0.20**	0.19*	0.13	0.02	0.12	0.00	0.29***	
Alcohol	M	−0.01	−0.15	−0.11	−0.19	−0.06	0.36	0.04	0.03
	F	0.06	−0.07	−0.15	−0.01	−0.15	0.20	0.16	−0.11

BMI: body weight index; PA: physical activity. Significance codes: 0 ‘***’ 0.001 ‘**’ 0.01 ‘*’ 0.05 ‘ ’ 1.

Table 3 shows results of linear regression analysis upon different adjustments of covariates. In unadjusted analysis, high smoking (≥7 cigarettes per day) was associated with increased TEE in men (regression coefficient [β] contrasting high smoking vs. never smoking = 356 kcal/day; p = 0.031), but not in women (β = 172; p = 0.191). This association was slightly mitigated with adjustment for the site of the study and adjustment for age did not substantially alter regression coefficients. In models adjusted for body weight and all other considered covariates (study site, alcohol intake, PA, and manual occupation), high smoking was associated with TEE in men (β = 298; p = 0.045) but not in women (β = 162; p = 0.170). In analysis adjusted for the same covariates but using FFM instead of body weight, the association between high smoking and TEE was slightly decreased in men (β = 245; p = 0.058) but largely attenuated in women (β = −76; p = 0.380).

**Table 3:** **Association between the differences in total energy expenditure (TEE) between smokers and non smokers according to smoking intensity and sex**

		Men (n = 104)			Women (n = 132)			All (n = 236)		
Adjustment*	Smoking	β	SE	P	β	SE	P	β	SE	P
None	Low	90	134	0.503	24	125	0.851	57	89	0.522
	High	356	163	0.031*	172	131	0.191	281	103	0.007*
Sites	Low	83	121	0.492	−56	126	0.660	1	88	0.987
	High	299	151	0.052	114	135	0.402	157	105	0.134
Sites, age	Low	82	122	0.504	−48	125	0.696	−2.8	87	0.974
	High	296	153	0.057	115	130	0.392	148	103	0.156
Sites, age, weight	Low	163	108	0.134	86	105	0.417	121	75	0.107
	High	327	134	0.017*	236	112	0.039*	260	88	0.003*
Sites, age, fat-free mass	Low	132	94	0.163	22	76	0.774	73	60	0.226
	High	290	118	0.016*	−5	82	0.949	155	71	0.031*
Sites, age, weight, alcohol, occupation, PA	Low	132	108	0.228	31	106	0.774	80	74	0.282
	High	298	146	0.045*	162	117	0.170	215	93	0.022*
Sites, age, fat-free mass, alcohol, occupation, PA	Low	94	94	0.316	−21	78	0.788	37	60	0.544
	High	245	127	0.058	−76	87	0.380	105	76	0.170

*The model "All" is adjusted for sex in addition to the other covariates.

β: regression coefficient; SE: standard error: PA: physical activity.

## Discussion

Our results indicate an association between smoking a substantial number of cigarettes and increased TEE in men, but not in women. Although these findings may reflect a true effect of smoking on TEE, the sex discordant findings may alternatively suggest a role of unaccounted potential confounding variables in men.

Our results are partially consistent with two previous population-based epidemiological studies of comparable design using the same DLW method. Specifically, while we found a significant increase in TEE among male smokers, the other two studies did not; however, numerical outcomes were similar. Bradley et al. [17] did not find a significant difference in TEE between smokers and non-smokers, but TEE was numerically greater (115 kcal/day) in men who smoked in analysis adjusted for age and FFM, hence their results are quite similar to ours. Warwick and Baines [25] did not report results adjusted for FFM, but TEE was greater by 334 kcal/day in smokers than in non-smokers in unadjusted analyses (but the difference was not statistically significant), hence again a result similar to ours. The absence of an effect of smoking on TEE at the statistical significance cut off of 0.05 in these two other studies is probably due to low statistical power. There were only 20 male smokers in the Bradley et al. study and a total of 10 male and female smokers in the Warwick and Baines study (compared to 57 male smokers and 21 female smokers in our study). Overall, results of these two other studies are consistent with our finding of a significant increase in TEE among male smokers of 100 to 300 kcal/day after essential adjustment for age and FFM [32]. Similar to the two other DLW studies, our study was likely underpowered to adequately assess the association among women, as further discussed below.

There was somehow less consistency about the association between TEE and smoking among women in the different DLW studies. Bradley et al. also found that the numerical difference in TEE between smokers and non-smokers was smaller in women than men. In contrast, the Warwick and Baines study shows a similar effect of smoking on TEE in men and women [25]. Even more than for men, statistical power is a problem underlying different conclusions between studies in view small numbers of female smokers (21 in our study, 27 in the Bradley study and 6 in the Warwick study). Importantly, it should also be reminded that smoking was not categorized by intensity in these two prior studies [17, 25]. This is an important limitation in these two other studies if we assume a dose response effect of smoking on TEE. In our study, only the “high” category of smoking (7 cigarettes per day or more; admittedly still not a very stringent cut off) was associated with 12% higher TEE.

The observed association between high smoking and increased TEE in men, but not in women, might be related to different factors. Women tend to smoke different cigarette brands than men and brands targeting women may contain different tobaccos and other products including appetite stimulants [33]. Alternatively, unaccounted cultural or other gender differences in the studied populations might also play a role. Given that the large majority of female smokers came from the US and South Africa, the unbalanced proportion of subjects from each study site might also have affected the results in women.

With regards to the effect of measured covariates on the association between smoking and TEE, adjustment for study site slightly impacted the association between smoking and TEE in men. This might reflect country-specific differences in either temperature, composition of cigarettes (e.g. different ingredients, nicotine levels or other additives), or some systematic reporting error. Also, smokers might walk longer distances to buy cigarettes and/or going to smoking areas in some countries. Adjustment for age did not impact the association between TEE and smoking. Adjustment for FFM vs. body weight had no substantial impact in men (i.e. the regression coefficients were of similar magnitude in models adjusted for weight or fat free mass) but this did have a large impact in women: adjustment for weight reinforced the association between TEE and smoking in women observed in crude analysis, while adjustment for FFM instead of weight made the association between smoking and TEE disappear. Adjustment for FFM vs. body weight has been considered to be essential when assessing associations with TEE [32]. This suggests that smoking is directly associated with FFM per unit of weight in women and, indeed, FFM (in kg) increased from 45.4 (±7.7), 45.3 (±5.6), to 50.4 (±5.6) in never, light and high smokers, respectively (p for trend = 0.074). Other adjustments for potential confounders such as alcohol, manual type of occupation and total physical activity did not substantially modify the association between smoking and TEE.

Several mechanisms can possibly account for differences in energy expenditure between smokers and never smokers. A few experimental studies investigated the effect of smoking on energy expenditure using a before/after type of design in same individuals. The comparison of our results with these studies is not straightforward because of differences in study designs. However, our results in men (12% higher TEE in smokers) are directionally consistent with two experimental studies in men, which found an increase of 3%–6% in energy expenditure at rest [20, 21]. Two experimental studies including male and female participants found a 3% increase in resting metabolic rate (RMR) after 30 minutes of smoking [22]. However RMR only accounts for 40 to 60% of TEE and a 3% increase in TEE would not be sufficient to account for all of the 12% higher TEE in male smokers than in never smokers observed in our study. In a cross-over calorimeter study on 4 men and 4 women, smoking resulted in a 10% increase in 24 hour TEE using a metabolic chamber, a method that captures all of basal resting metabolic rate, thermic effect of eating and physical activity, albeit under restrained space conditions that are not conducive to normal voluntary PA [19]. This experimental result is consistent with our results in men. With regards to our null finding in women when adjusting for FFM, a study done in 13 female heavy smokers who quit smoking for 48 days did no report a change in RMR before and after quitting [23]. Taken altogether, these experimental studies suggest that TEE is increased when measured shortly after smoking but there is less evidence when TEE is measured after extended periods of quitting.

With regards to mechanisms underlying smoking and TEE; smoking may increase RMR, e.g. by increasing circulating catecholamines, which in turn would result in increased TEE and decreased BMI. As mentioned in the introduction of this paper, this mechanism is supported by some studies [19–22] but not by others [17, 23, 25]. Alternatively, smoking could be associated with lower food intake, resulting in lower TEE and lower BMI. However, studies on this question have produced inconsistent results, likely due to known low accuracy of self-reported dietary data [17]. Yet, an experimental study showed that nicotine administration decreased both hunger and food intake during the following two hours of administration [34]. Documents suggest that the tobacco industry has been adding appetite-suppressant substances into cigarettes [35], which could result in decreased energy intake and decreased BMI. A combination of these smoking effects in opposite directions is also possible (increased RMR and decreased appetite), which could result in decreased BMI and increased TEE. Of note, the increase in BMI generally found after smoking cessation is a partially distinct issue and other mechanisms may also be involved, including increased food intake as a reactive behavior to mimic gestures of smoking cigarettes or to cope with nicotine withdrawal [36].

We found that body weight was 3-5% lower in smokers than in never smokers (not stastically significant; p <0.05), in contrast with a larger effect in numerous studies, often conducted many years ago [10–13], but in accordance with small or null effect in some recent studies [14, 15]. Several reasons may explain these discrepancies. First, our sample size was small and insufficiently powered to detect small BMI differences. Second, smokers in our study smoked a fairly small number of cigarettes per day (including within our category of “high” smokers), which limits the potential to detect a small effect of smoking on BMI if the relation is dose-dependent. Third, participants in our study were of African origin, whereas a difference was often noted in studies including mainly Caucasian populations. The populations’ metabolic responses subsequent to smoking might differ in persons of African vs. Caucasian origin [37]. Fourth, our study included a young population (≤45 years), in which the effect of smoking on BMI might not yet be fully apparent. Finally, as previously mentioned, a lower BMI in smokers vs. non smokers was mostly observed in studies in the 1980s and 1990s [10–13], with a trend toward lesser difference in several more recent studies [14, 15]. A decreasing difference in mean BMI according to smoking status over time could be due to the upward secular trend in BMI in populations worldwide [38], which could mask a leaning effect of smoking or reflect the downward secular trend in cigarette consumption in many countries. Also, smoking has shifted from high to low socio-economic status over time [39], along the shift of overweight (including among non smokers) from high to low socio economic status persons: these parallel secular trends might mitigate an effect of smoking on TEE in current population-based studies. More generally, differences in BMI between smokers and never smokers may be attenuating over time because of factors not related to the hypothesized direct effect of smoking on weight. Further research on this important question for public health is warranted.

The main limitations of our study are that it included mostly light or moderate smokers (inclusive in our “high” smoking category) and the numbers of smokers were fairly small, especially among women, reducing the statistical power to detect an effect of smoking on TEE and on BMI, especially in women. Also, CO_2_ inhaled through smoking could potentially artificially increase TEE calculated by the DLW technique, but this effect has been estimated to be very small [17]. Finally, a causal link between smoking and TEE or BMI cannot be inferred from cross-sectional data.

## Conclusions

There was an association between smoking and TEE among men. However, the lack of an association among women when adjusting for FFM, which may be partly related to the small number of smoking females, is also consistent with unaccounted confounding factors. The effects of smoking on TEE, and indirectly on body weight, have important implications for public health programs. Therefore, further studies with accurate measurements of TEE, physical activity and caloric intake (food assessment surely is the largest challenge in view of limited accurate instruments) and large enough sample sizes are still needed to provide definite results and help understand the underlying mechanisms.

## Abbreviations

BMI: Body mass index
r: Correlation coefficient
DLW: Doubly labeled water
FFM: Fat-free mass
METS: The modeling the epidemiological transition study
PA: Physical activity
β: Regression coefficient
RMR: Resting metabolic rate
SE: Standard error
TBW: Total body water
TEE: Total energy expenditure
USA: United States of America.
